# KML001 Induces Apoptosis and Autophagic Cell Death in Prostate Cancer Cells via Oxidative Stress Pathway

**DOI:** 10.1371/journal.pone.0137589

**Published:** 2015-09-09

**Authors:** Dalsan You, Yunlim Kim, Myoung Jin Jang, Chunwoo Lee, In Gab Jeong, Yong Mee Cho, Jung Jin Hwang, Jun Hyuk Hong, Hanjong Ahn, Choung-Soo Kim

**Affiliations:** 1 Department of Urology, Asan Medical Center, University of Ulsan College of Medicine, Seoul, Korea; 2 Asan Institute for Life Sciences, Asan Medical Center, University of Ulsan College of Medicine, Seoul, Korea; 3 Department of Pathology, Asan Medical Center, University of Ulsan College of Medicine, Seoul, Korea; 4 Institute for Innovative Cancer Research, Asan Medical Center, University of Ulsan College of Medicine, Seoul, Korea; University of Manitoba, CANADA

## Abstract

We investigated the effects of KML001 (NaAsO_2_, sodium metaarsenite, Kominox), an orally bioavailable arsenic compound, on the growth and death of human prostate cancer cells and its mechanism of action. Growth inhibition was assessed by cytotoxicity assays in the presence or absence of inhibitor of apoptosis, inhibitor of autophagy or antioxidant *N*-Acetyl-_L_-cysteine to study mechanism of cell death induced by KML001 in PC3, DU145 and LNCaP prostate cancer cell lines. Electron microscopy, flow cytometry and Western blotting were used to study apoptotic and autophagic mechanisms. The DU145 xenograft model was used to determine the efficacy of KML001 in vivo. KML001 decreased the viability of cells and increased the percentage of annexin V-positive cells dose-dependently in prostate cancer cells, and LNCaP cells were more sensitive to KML001 than PC3 or DU145 cells. Electron microscopy revealed typical apoptotic characters and autophagic vacuoles in cells treated with KML001. Exposure to KML001 in prostate cancer cells induced apoptosis and autophagy in a time- and dose-dependent manner. KML001 induced dose-dependent accumulation of reactive oxygen species, and scavenging the reactive oxygen species with *N*-Acetyl-_L_-cysteine reduced LC3 and cleaved poly (ADP-ribose) polymerase. KML001 significantly inhibited tumor growth in the DU145 xenograft model. In addition, significant decrease of proliferation and significant increases of apoptosis and autophagy were observed in KML001-treated tumors than in vehicle-treated tumors. Exposure of human prostate cancer cells to KML001 induced both apoptosis and autophagic cell death via oxidative stress pathway. And KML001 had an antiproliferative effect on DU145 cells in xenograft mice.

## Introduction

While arsenic compounds are widely known as carcinogens that induce cancers in many human tissues, arsenic trioxide (As_2_O_3_, ATO) has been demonstrated clinically to be an effective therapeutic agent for the treatment of acute promyelocytic leukemia [1,2]. Although its anticancer mechanism of action is not well understood, ATO has been found to regulate various biological functions, including cell proliferation, apoptosis, differentiation, and angiogenesis in various cell lines [3]. Because ATO was successful in treating acute promyelocytic leukemia [1,2,4], arsenicals are experiencing a revival in modern cancer medicine [5].

Arsenic exists in tri- and penta-valent oxidation states as chemically unstable sulfide and oxide, and as salts of sodium, potassium, or calcium. Trivalent arsenicals, including KML001 (NaAsO_2_, sodium metaarsenite, Kominox) and ATO, inhibit many enzymes by reacting with biological ligands that have free sulfur groups [3,6]. Three major molecular mechanisms of ATO-induced apoptosis have been evaluated, involving mitogen-activated protein kinases, caspases, and reactive oxygen species **(**ROS) [3]. Although the mechanism of action of ATO is well-known [7–12], that of KML001 is still under investigation [13,14].

Due to its oral bioavailability, water solubility and lower median lethal dose (LD_50_) in rats, KML001 is more suitable for clinical applications than ATO [13]. We therefore investigated the ability of KML001 to inhibit the growth and induce cell death of human prostate cancer cell lines. We also analyzed whether autophagy is with apoptosis involved in KML001-induced cell death in prostate cancer cell lines. Finally, we determined the antitumor effect of KML001 in DU145 xenograft model.

## Materials and Methods

### Cell lines and reagents

The human prostate cancer cell lines, PC3, DU145, and LNCaP were purchased from ATCC (Manassas, VA, USA) and maintained in RPMI 1640 (Invitrogen, Carlsbad, CA, USA) with 10% heat inactivated fetal bovine serum (FBS), 100 units/ml of penicillin, and 100 μg/ml of streptomycin in a 5% CO_2_ atmosphere at 37°C. KML001 was obtained from Komipharm International (Gyeonggi-do, Korea). Z-VAD-FMK was purchased from R&D systems (Minneapolis, MN, USA). 3-*methyl*-adenine (3-MA) and *N*-Acetyl-_L_-cysteine (NAC) were purchased from Sigma (St. Louis, MO, USA). The drug treatment protocol was described previously [15].

### Measurement of cell proliferation and viability

Cell proliferation was assessed using Alamar blue (AbD Serotec, Kidlington, Oxford, UK) according to the manufacturer’s instructions and performed as described previously [15]. Cell viability assay was measured using Celltiter Glo Luminescent Cell Viability Assay (Promega, Madison, WI, USA) and performed as described previously [15]. IC_50_ were calculated using GraphPad Prism version 5.00 (GraphPad Software, San Diego, CA, USA).

### Electron microscopy

Human prostate cancer cells were treated with IC_50_ concentrations of KML001 for 24 and 48 h. Cells were treated as described previously [15], and photographed using a transmission electron microscope (JEOL model 1200EX, Tokyo, Japan).

### Assays for apoptosis detection

Human prostate cancer cells were exposed to KML001 and apoptosis was assessed by flow cytometry using the annexin V-FITC Apoptosis Detection Kit (BD Biosciences, Bedford, MA, USA) according to the manufacturer’s instructions, and analyzed as described previously [15].

### Western blot analysis

Human prostate cancer cells cultured in RPMI 1640 with 5% heat inactivated FBS were treated with KML001 at various concentrations for different time periods, followed by incubation for 6 h with and without Z-VAD-FMK (20 μM) or 3-MA (2 mM). Western blot analysis was performed as described previously [15]. Antibodies to LC3 (Novus Biologicals, Littleton, CO, USA), procaspase-3, and poly (ADP-ribose) polymerase (PARP, Cell Signaling Technology, Danvers, MA, USA), and β-actin (Santa Cruz Biotechnology, Santa Cruz, CA, USA) were used for Western blot analysis.

### ROS detection

Intracellular ROS generated by KML001 or hydrogen peroxide (H_2_O_2_) as a positive control were measured using an assay based on the intracellular peroxide dependent oxidation of 2',7'-dichlorodihydrofluorescein diacetate (DCFH-DA; Molecular Probes, Eugene, OR, USA) to the fluorescent compound 2',7'-dichlorofluorescein (DCF), and analyzed as described previously [16].

### DU145 xenograft animal model

All aspects of animal care and treatment were performed according to the eighth edition of the Guide for the Care and Use of Laboratory Animals published in 2011. The protocol was approved by Institutional Animal Care and Use Committee of Asan Medical Center, Seoul, Korea (2012–02–067). Four-week-old male BALB/C nude mice (OrientBio, Seoul, Korea) were subcutaneously inoculated with 5 × 10^6^ DU145 cells. When the tumors reached an average volume of about 100 mm^3^, the mice were randomly divided into control and treatment groups (6 animals per group). For the DU145 bearing mice, the treatment groups were administered KML001 (2.5 or 10 mg/kg/d) and daily by oral gavages for 4 weeks. Mice were monitored for toxicity by body weight measurements, and tumors were measured three times a week and volume was calculated by the modified ellipsoid formula: 0.52×length×(width)^2^ [17]. Mice were euthanized by carbon dioxide after harvesting tumors.

### TUNEL assay and Ki-67 immunohistochemical staining

Terminal deoxynucleotidyl transferase (TdT)-mediated dUTP nick end labelling (TUNEL) for the detection of apoptotic cells were performed using the *in situ* cell death detection kit (Roche Molecular Biochemicals, Mannheim, Germany) according to the manufacturer's protocols. For image analysis, three randomly selected fields from each mice were photographed at 200× magnification using fluorescence microscopy (Carl Zeiss, Jena, Germany).

For Ki-67 immunostaining using proliferation markers, tissue paraffin-embedded sections were deparaffinized using xylenes and graded ethanol followed by antigen retrieval using IHC-Tek epitope retrieval steamer set (IHC World, LLC. Woodstock, MD, USA). Slides staining were performed as described previously [18]. For images and intensity scoring, three randomly selected fields from each mice were photographed at 200× magnification using an Olympus U-LH100L-3 camera and Olympus Ix 71 software.

### Statistical Analysis

All data were shown as the means ± standard deviations (SD). Statistical significance was considered at *p* < 0.05 and determined by one-way analysis of variance (ANOVA).

## Results

### Effect of KML001 on proliferation of prostate cancer cells

**Fig 1:**
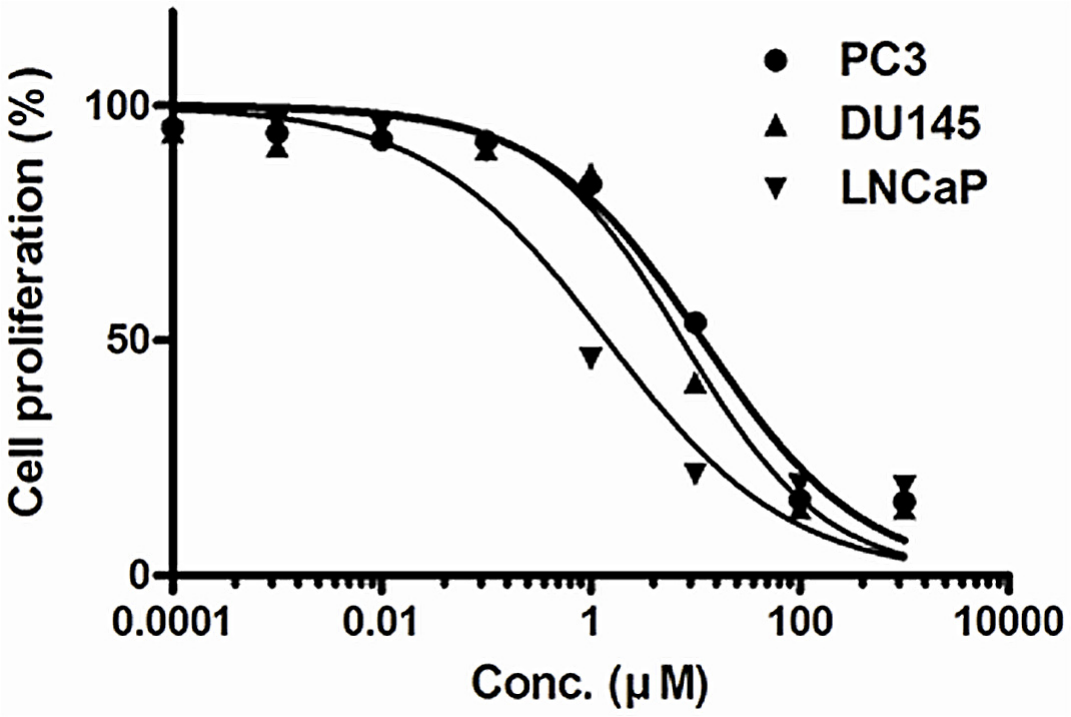
Growth inhibition of KML001 in prostate cell lines. Cells treated with various concentrations of KML001 were incubated for 72 h. The Alamar blue assay was done in triplicate. Mean values are given, the value of control being 100%. PC3 (●, *thick solid line*), DU145 (▲, *thin solid line*), and LNCaP (▼, *thin solid line*).

Incubation of androgen-independent PC3 and DU145 and androgen-dependent LNCaP prostate cancer cells with 0.0001–200 μM KML001 for 72 h reduced cell viability in a dose-dependent manner. LNCaP cells were more sensitive than PC3 or DU145 cells (Fig 1; Table 1).

**Table 1:** IC_50_ values of KML001 in prostate cancer cells.

Prostate cancer cells	IC_50_ (μM)	Range	Rank IC_50_
PC3	11.85	9.38–14.97	3
DU145	7.57	5.78–9.91	2
LNCaP	1.45	0.96–2.19	1

### Induction of apoptotic cell death

**Fig 2:**
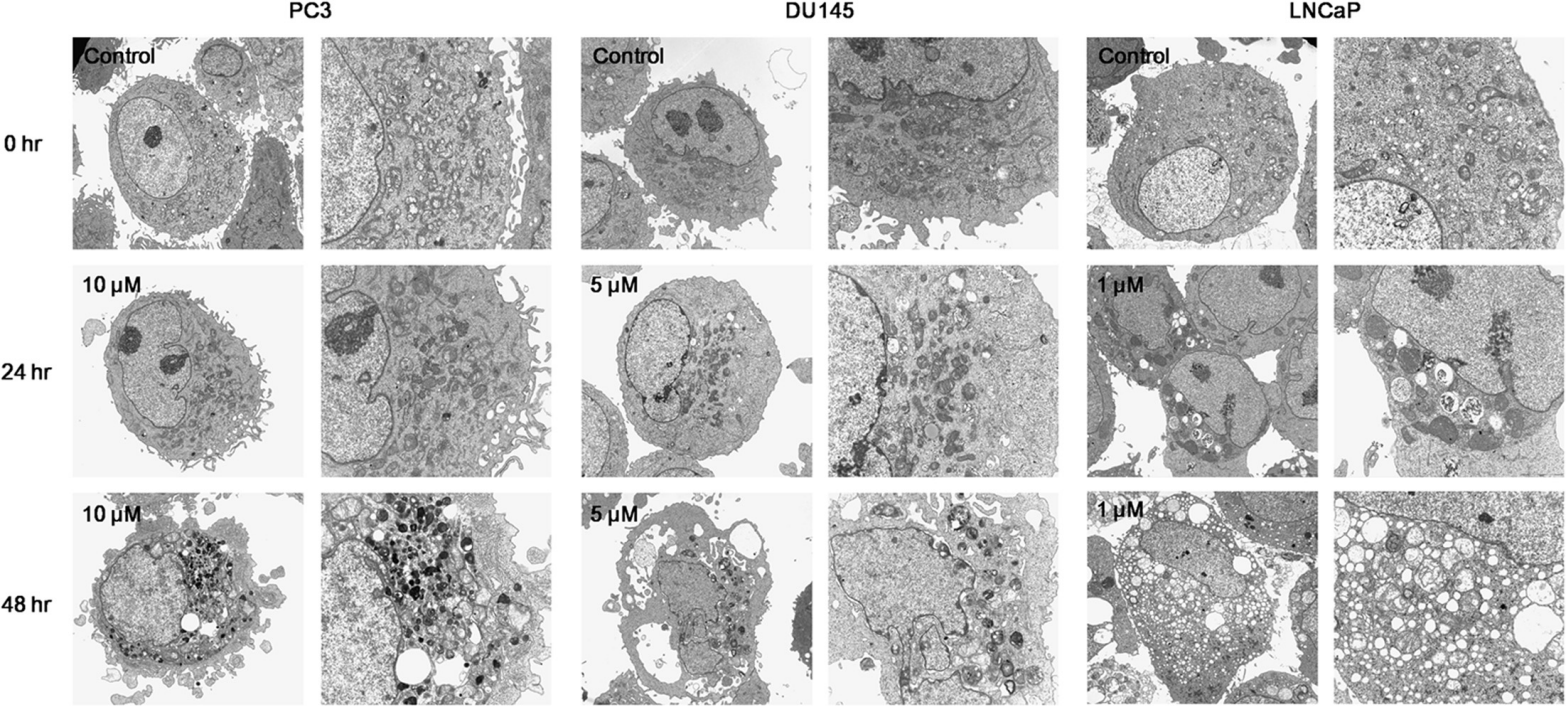
Structural features observed by electron microscope (10000× and 5000×) in PC3, DU145, and LNCaP prostate cancer cells treated with KML001 for 24 and 48 h.

To determine whether treatment with KML001 (10 μM) induces apoptosis, the ultrastructure of KML001-treated PC3 cells was analyzed by electron microscopy. PC3 cells treated with KML001 showed typical apoptotic characters, including a smaller nucleus, more concentrated cytoplasm, crumpled nuclear membrane, and chromosomes condensed into a semilunar shape with attachments to the nuclear and cellular membranes. Moreover, apoptotic bodies were observed when the chromatin condensed and ruptured to the nuclear margin. Similar results were observed in DU145 and LNCaP cells treated with IC_50_ concentrations of KML001 (5 μM and 1 μM, respectively) (Fig 2).

**Fig 3:**
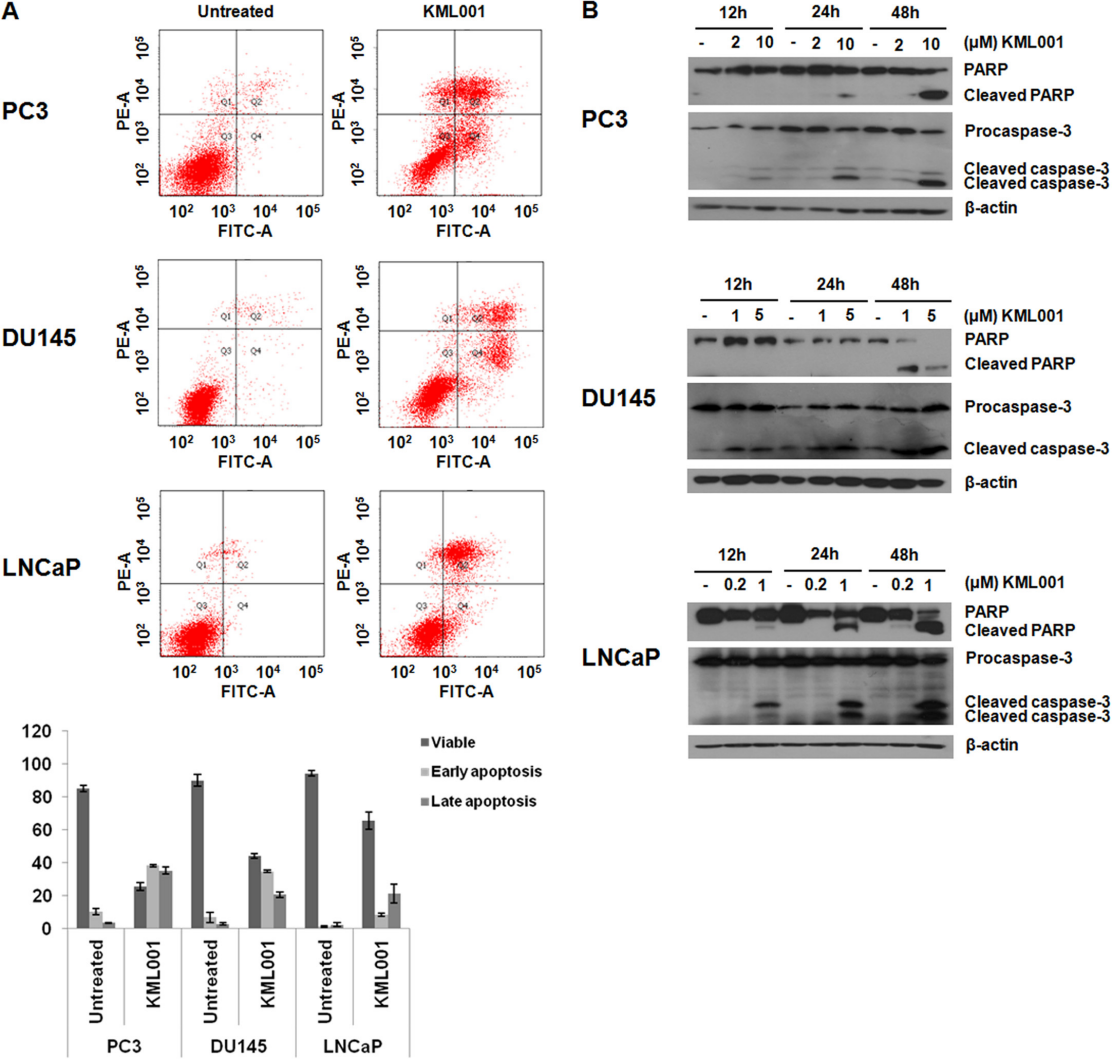
Induction of apoptosis by KML001 in prostate cancer cells. (A) FACS analysis of annexin V/PI staining. Results show early apoptosis, defined as annexin V-positive and PI-negative cells, and late apoptosis, defined as annexin V-positive and PI-positive cells. Results were expressed as means ± SD of three independent experiments. (B) Western blot analysis of the time- and dose-dependent cleavage of PARP and activation of procaspase-3.

To investigate the mechanism of KML001-induced cell death, we performed annexin V flow cytometry assays. Treatment of these cells with KML001 yielded cells positive for annexin V staining only and cells positive for annexin V and PI staining. These results indicate that KML001 induced cell death via both apoptosis and necrosis (Fig 3A).

We also found that exposure of prostate cancer cells to KML001 resulted in a time- and dose-dependent increase in the cleavage of PARP, as well as the activation of procaspases-3, which mediates intrinsic apoptosis (Fig 3B). To confirm the role of apoptosis in the KML001-induced death of prostate cancer cells, we treated the cells with the caspase inhibitor Z-VAD-FMK. We found that addition of 20 μM Z-VAD-FMK decreased the activation of procaspases-3 in all prostate cancer cell lines (data not shown), supporting the hypothesis that KML001 induced apoptosis in prostate cancer cells.

### Induction of autophagic cell death

To determine whether treatment with KML001 (10 μM) induces autophagy, the ultrastructure of PC3 treated cells was examined by electron microscopy. We observed autophagic vacuoles, including autophagosomes or autolysosomes, in KML001-treated PC3 cells, as well as in DU145 and LNCaP cells treated with their respective IC_50_ concentrations of KML001 (Fig 2).

**Fig 4:**
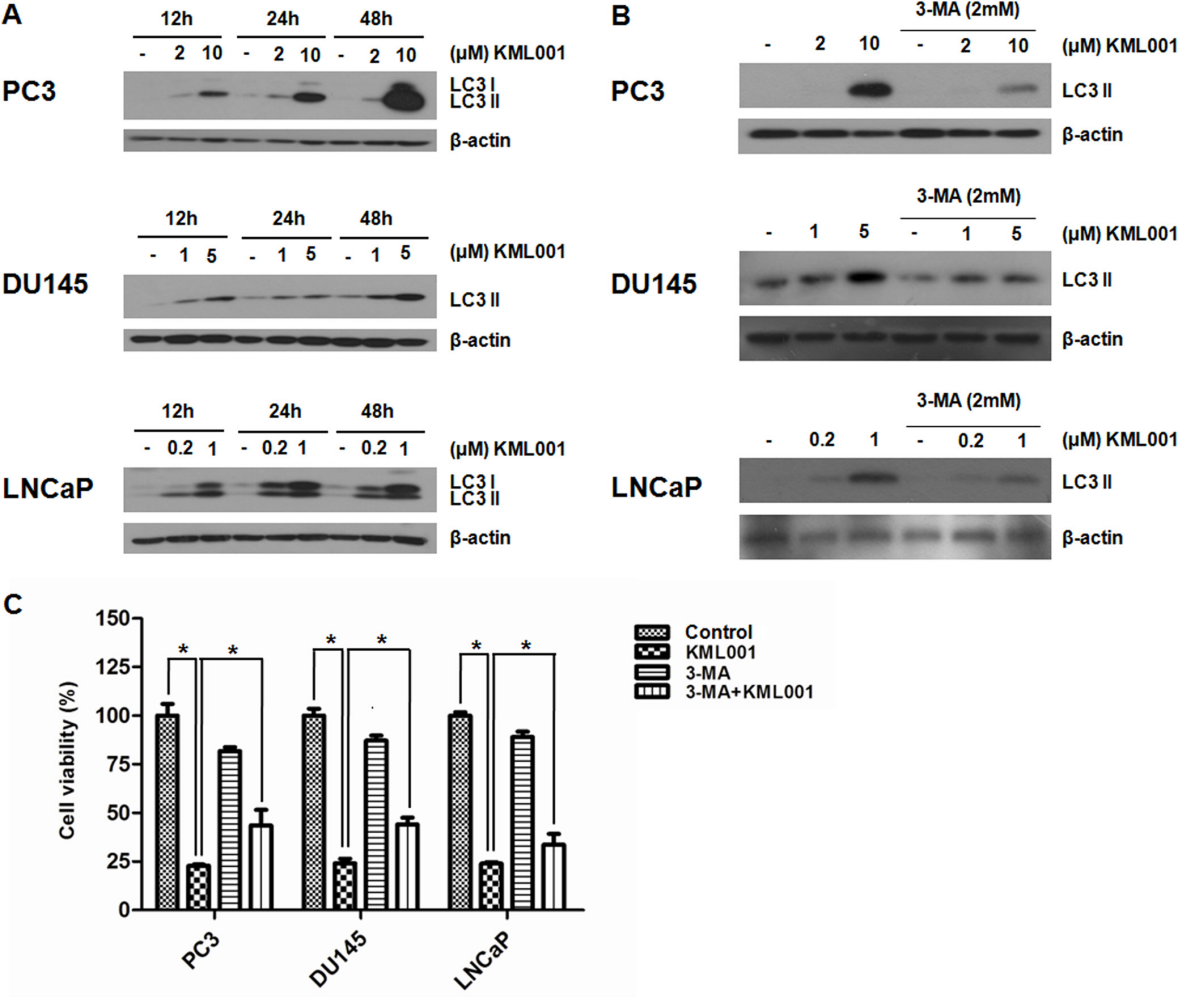
Induction of autophagy by KML001 in prostate cancer cells. (A) Western blot analysis of the time- and dose-dependent conversion of LC3-I to-II. (B) Inhibition by 3-MA of KML001-induced conversion of LC3 in prostate cancer cells. (C) Cells were exposed to 10 μM (PC3), 5 μM (DU145), or 2 μM (LNCaP) KML001 in the presence or absence of 1 mM 3-MA for 72 h. Results were expressed as means ± SD of three independent experiments. * *p* < 0.05 by one-way ANOVA.

Prostate cancer cells exposed to KML001 were examined by Western blotting, using an anti-LC3 antibody that recognizes both forms of LC3. We found that expression of LC3 and/or conversion to LC3-II increased in a time- and dose-dependent manner, relative to untreated cells (Fig 4A). To confirm the role of autophagy in the KML001-induced death of prostate cancer cells, we treated the cells with the autophagy inhibitor 3-MA. We found that addition of 2 mM 3-MA decreased the expression of LC3-II in all prostate cancer cell lines (Fig 4B). In addition, addition of 1 mM 3-MA also attenuated KML-induced cell death in all prostate cancer cell lines (Fig 4C). Taken together, these findings support hypothesis that KML001 induced autophagy-mediated cell death in prostate cancer cells.

### The antioxidant NAC protects cells from KML001-induced cell death

**Fig 5:**
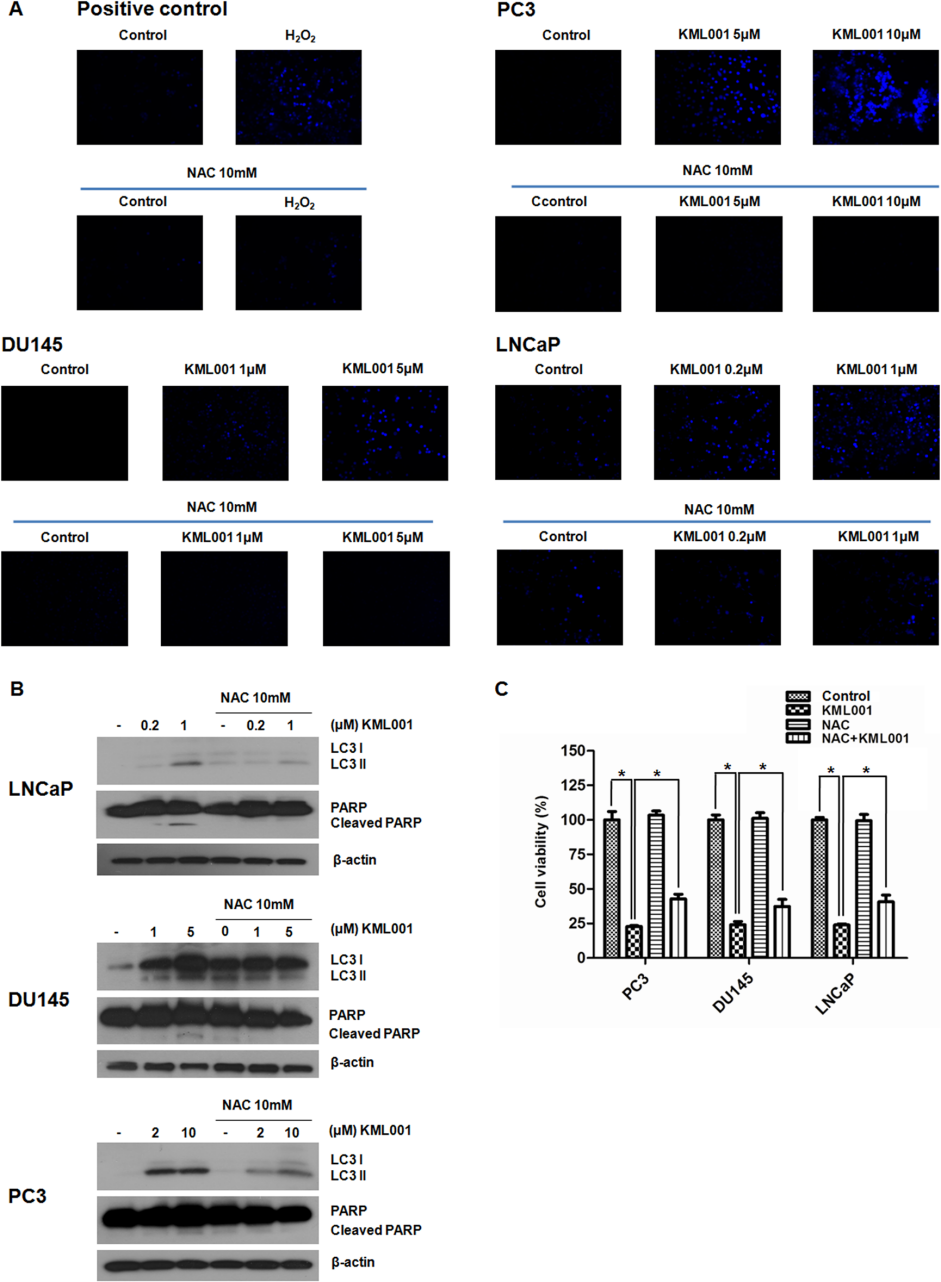
Regulation of autophagy and apoptosis by ROS. All 3 prostate cancer cells were treated with the indicated concentration of KML001 in the absence or presence of 5 mM NAC for 24 h. (A) KML001 induces dose-dependent ROS (blue) accumulation. Cells were stained with DCFH-DA and washed with PBS. More than three fields in each cell were observed by fluorescence microscope (200×), and representative images are shown. (B) NAC inhibition of KML001-induced conversion of LC and caspase activation in prostate cancer cells. (C) Cells were exposed to 10 μM (PC3), 5 μM (DU145), or 2 μM (LNCaP) KML001 in the presence or absence of 1 mM NAC for 72 h. Results were expressed as means ± SD of three independent experiments. * *p* < 0.05 by one-way ANOVA.

KML001 induced dose-dependent ROS accumulation in all 3 prostate cancer cell lines (Fig 5A). We also examined the effects of the antioxidant NAC on KML001-induced apoptosis and autophagy. We found that treatment of all 3 prostate cancer cell lines with 1 mM NAC decreased the expression of LC3-II and the proteolysis of PARP (Fig 5B). In addition, addition of 1 mM NAC also attenuated KML-induced cell death in all prostate cancer cell lines (Fig 5C). These findings provide further evidence that exposure of prostate cancer cells to KML001 activated both apoptosis and autophagy via oxidative stress.

### Effect on DU145 xenografts

**Fig 6:**
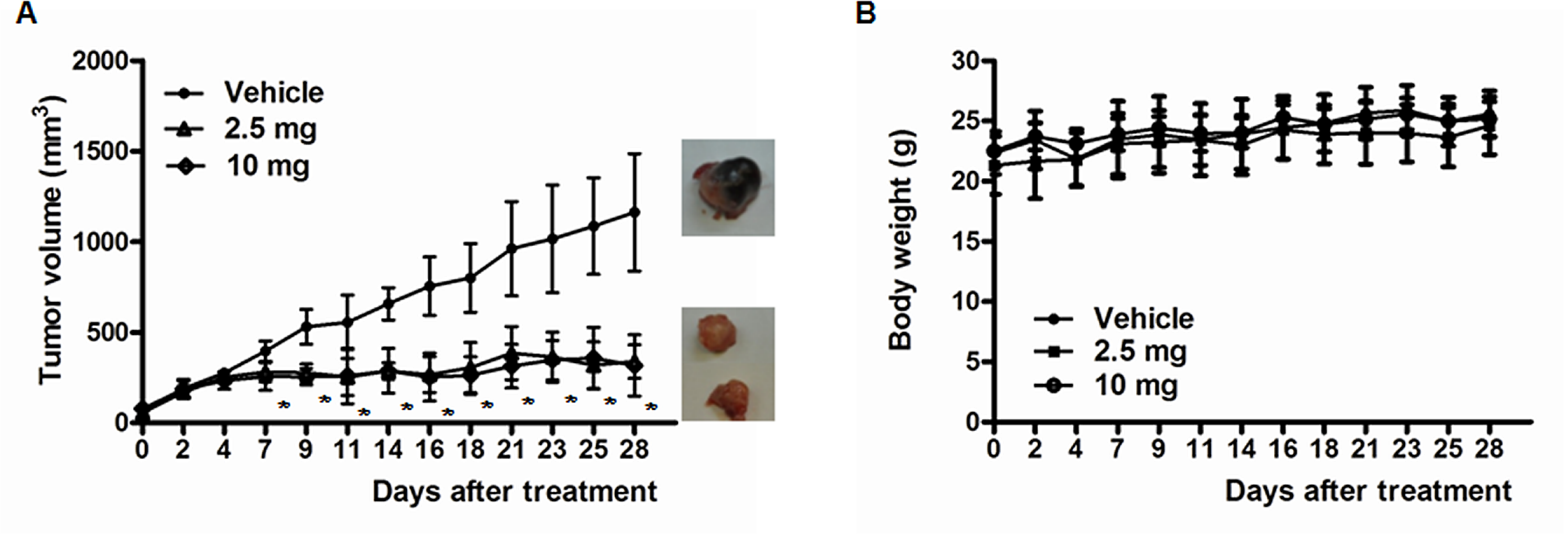
KML001 treatment has (A) a growth inhibitory effect on DU145 prostate cancer cells in mice, and (B) no effect on body weight of mice. Vehicle and KML001 group mice orally received saline and KML001 (2.5 or 10 mg/kg/d) for 4 weeks, respectively. * *p* < 0.05 vs. vehicle.

We subcutaneously injected nude mice with 5 × 10^6^ DU145 cells to test the effects of KML001 on androgen-independent prostate cancer cells in vivo. After the tumor size reached 100 mm^3^, we administered KML001 (2.5 or 10 mg/kg/d) to them for 4 weeks. In the DU145 xenograft model, tumor growth was inhibited with KML001 as compared to vehicle (Fig 6A). But KML001 treatment had no effect on body weight of mice (Fig 6B)

**Fig 7:**
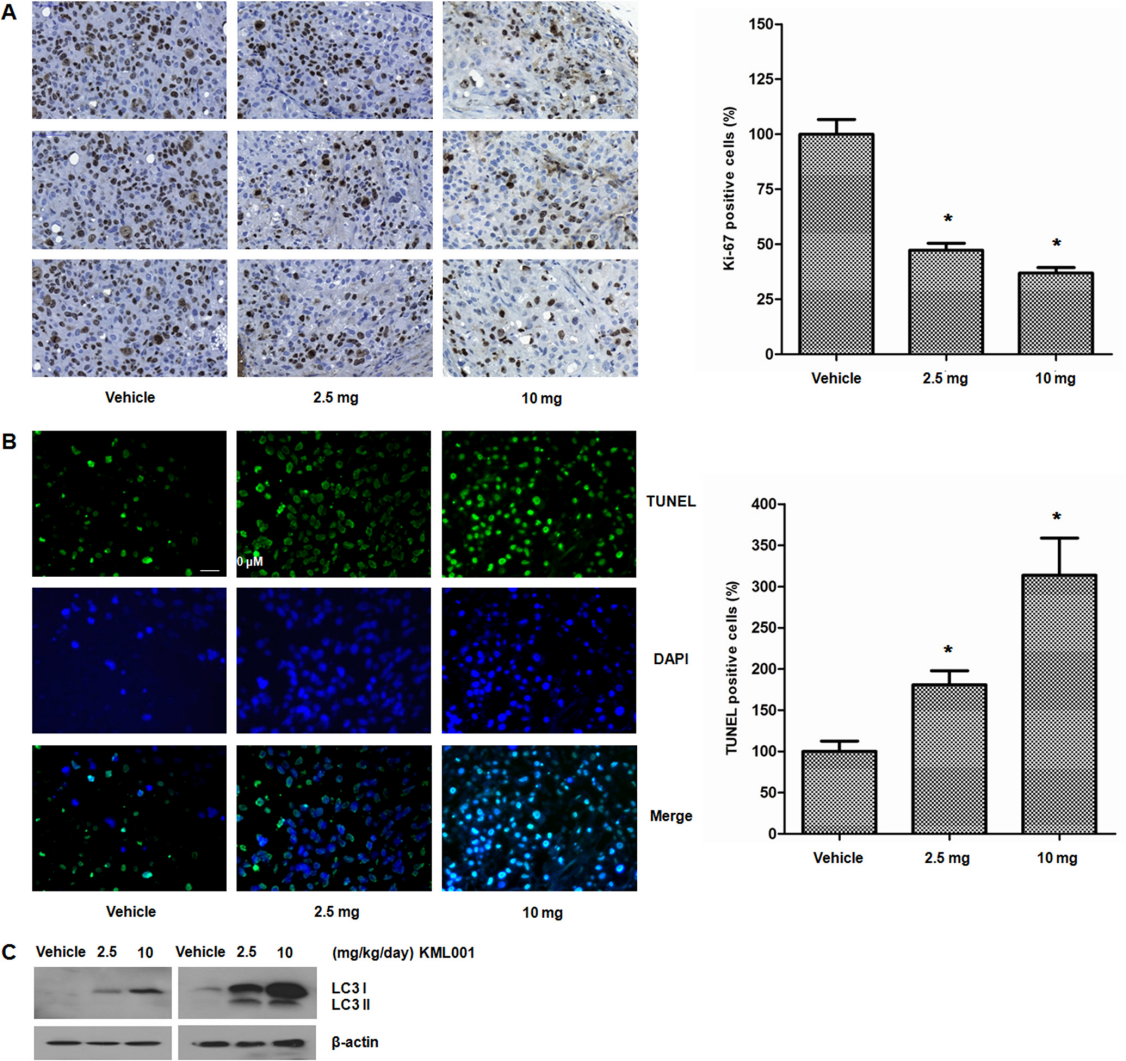
KML001 treatment has (A) anti-proliferative effect, (B) apoptotic effect, and (C) autophagic effect on DU145 prostate cancer cells in mice. Three fields in each mice were observed by fluorescence (200×) and bright field microscopes (200×), respectively. Representative images of each treatment group were shown. Vehicle and KML001 group mice orally received saline and KML001 (2.5 or 10 mg/kg/d) for 4 weeks, respectively. * *p* < 0.05 vs. vehicle.

In the next experiment, we determined the effect of KML001 on proliferation, apoptosis, and autophagy in tumor tissues harvested from animals of different treatment groups. In immunohistochemical staining of the proliferation marker Ki-67, significant decreases in Ki-67 immunostaining were observed in KML001-treated tumors than in vehicle-treated tumors (Fig 7A). In TUNEL assay, significant greater apoptotic cells were observed in KML001-treated tumors than in vehicle-treated tumors (Fig 7B). In Western blot analysis, we found that expression of LC3 and/or conversion to LC3-II increased in a dose-dependent manner, relative to vehicle-treated tumors (Fig 7C).

## Discussion

Prostate cancer is one of the leading causes of death among men in the United States [19]. Although aggressive efforts toward early detection and treatment decrease mortality rate for prostate cancer, prostate cancer is the second most common cause of cancer death among men yet. Although early-stage prostate cancer requires androgen for growth and thus responds to androgen deprivation therapy, the disease may progress to androgen-independence and may be unresponsive to androgen ablation [20]. Docetaxel-based chemotherapies have shown palliative and survival benefits for patients with castration-resistant prostate cancer, but treatment results are generally unsatisfactory with a median survival time of only 16 to 18 months [21,22]. So it is not only important to develop effective ways of preventing or slowing the formation of castration-resistant prostate cancer, but also to develop new chemotherapeutic agents for the treatment of castration-resistant prostate cancer.

Since ATO was shown to have dramatic effects in patients with acute promyelocytic leukemia [1,2], this agent has also been tested in patients with solid tumors, including prostate cancer [23]. Both ATO and KML001 are trivalent arsenicals and identical substances in solution, with similar cytotoxicity against androgen-independent prostate cancer cells [13]. However, the oral bioavailability and water solubility of KML001 suggest its clinical applicability, compared with ATO. Moreover, KML001 was shown to have higher LD_50_ (41.6 mg/kg) in rats, compared with ATO (14.6 mg/kg) [13]. So we tested the ability of KML001 to inhibit the growth and induce cell death of human prostate cancer cell. We found that KML001 treatment reduced the proliferation of all 3 prostate cancer cell lines, being more toxic to androgen-dependent LNCaP cells than to androgen-independent cells. More importantly, KML001 had an antiproliferative effect on androgen-independent DU145 cells in vitro and in vivo.

ATO acts on malignant cells through a variety of mechanisms, targeting multiple signal transduction pathways and resulting in induction of apoptosis, antiproliferative activity, and antiangiogenesis [3]. Several recent studies have reported that ATO and KML001 may target a telomere/telomerase complex [8–10,13]. KML001 binds to telomeric sequences and erodes telomere, resulting in telomere-associated DNA damage induction and telomere attrition. In addition, ATO was found to induce type II programmed cell death, autophagy, in malignant glioma cells [11,24]. We have shown here that KML001 treatment resulted in the formation of autophagic vacuoles, as documented by electronic microscopy. Moreover, KML001 induced a time- and dose-dependent increase in LC3-II. Using the autophagy inhibitor 3-MA, we corroborated that the mechanism of KML001-induced cell death involves autophagy. Autophagy is not only responsible for cell killing by itself, but also participates in a lethal signaling event inducing apoptosis or necrosis [25,26].

Additionally, we found that the activation of autophagy and apoptosis by KML001 was mediated by oxidative stress. ROS play a pivotal role in mediating the cytotoxicity induced by KML001. Several studies have found that ROS play important roles in regulating both normal cellular processes and disease progression [27–29]. In addition, accumulated ROS have been known as the key intermediate for the cytotoxicity induced by chemotherapeutic agents, including ATO [3].

One concern regarding the use of arsenic for clinical applications is its toxicity in humans. Clinical studies have shown that ATO at concentrations less than 2 μM does not induce severe side effects [30,31]. As mentioned above, KML001 was less toxic to rats at the same concentration of ATO [13]. In our study, KML001 had no adverse effect on body weight of DU145 xenograft mice. Our results therefore suggest that KML001 may be clinically useful in patients with castration-resistant prostate cancer.

Whereas most chemotherapeutic agents are aimed at inhibiting the growth of castration-resistant prostate cancer, androgen-dependent and-independent prostate cancer cells have been reported to be equally susceptible to ATO-induced apoptosis [23]. Moreover, inhibition by ATO was more pronounced in prostate cancer cells expressing androgen receptor than in prostate cancer cells depleted of androgen receptor, and inhibition of androgen receptor activity by ATO and by the androgen receptor antagonist, bicalutamide, was additive [24]. These results may warrant the future assessment of the effects of KML001, alone or in combination with androgen deprivation therapy, on the progression of androgen-dependent LNCaP to androgen independence in a nude mice xenograft model.

## Conclusions

We have shown here that exposure of androgen-dependent and-independent prostate cancer cells to KML001 activated both apoptosis and autophagic cell death through oxidative stress. In addition, KML001 had an antiproliferative effect on androgen-independent DU145 cells in vivo.
