# Correction: KML001 Induces Apoptosis and Autophagic Cell Death in Prostate Cancer Cells via Oxidative Stress Pathway

**DOI:** 10.1371/journal.pone.0225087

**Published:** 2019-11-06

**Authors:** Dalsan You, Yunlim Kim, Myoung Jin Jang, Chunwoo Lee, In Gab Jeong, Yong Mee Cho, Jung Jin Hwang, Jun Hyuk Hong, Hanjong Ahn, Choung-Soo Kim

There is an error in Fig 2, “Structural features observed by electron microscope (10000× and 5000×) in PC3, DU145, and LNCaP prostate cancer cells treated with KML001 for 24 and 48 h.” Please see the correct Fig 2 here.

**Fig 2:**
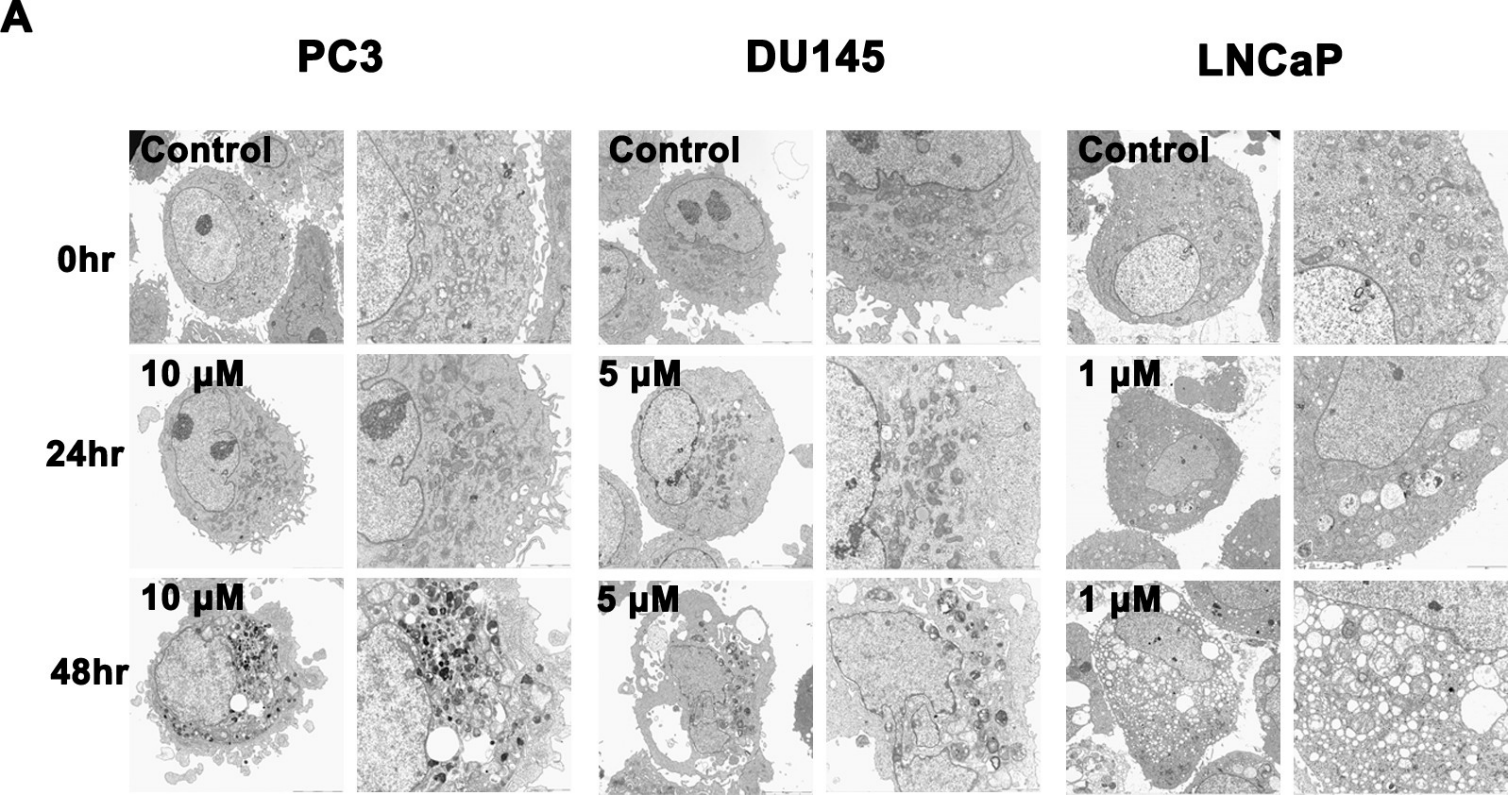
Structural features observed by electron microscope (10000× and 5000×) in PC3, DU145, and LNCaP prostate cancer cells treated with KML001 for 24 and 48 h.

There is an error in Fig 3, “Induction of apoptosis by KML001 in prostate cancer cells.” Please see the correct Fig 3 here.

**Fig 3:**
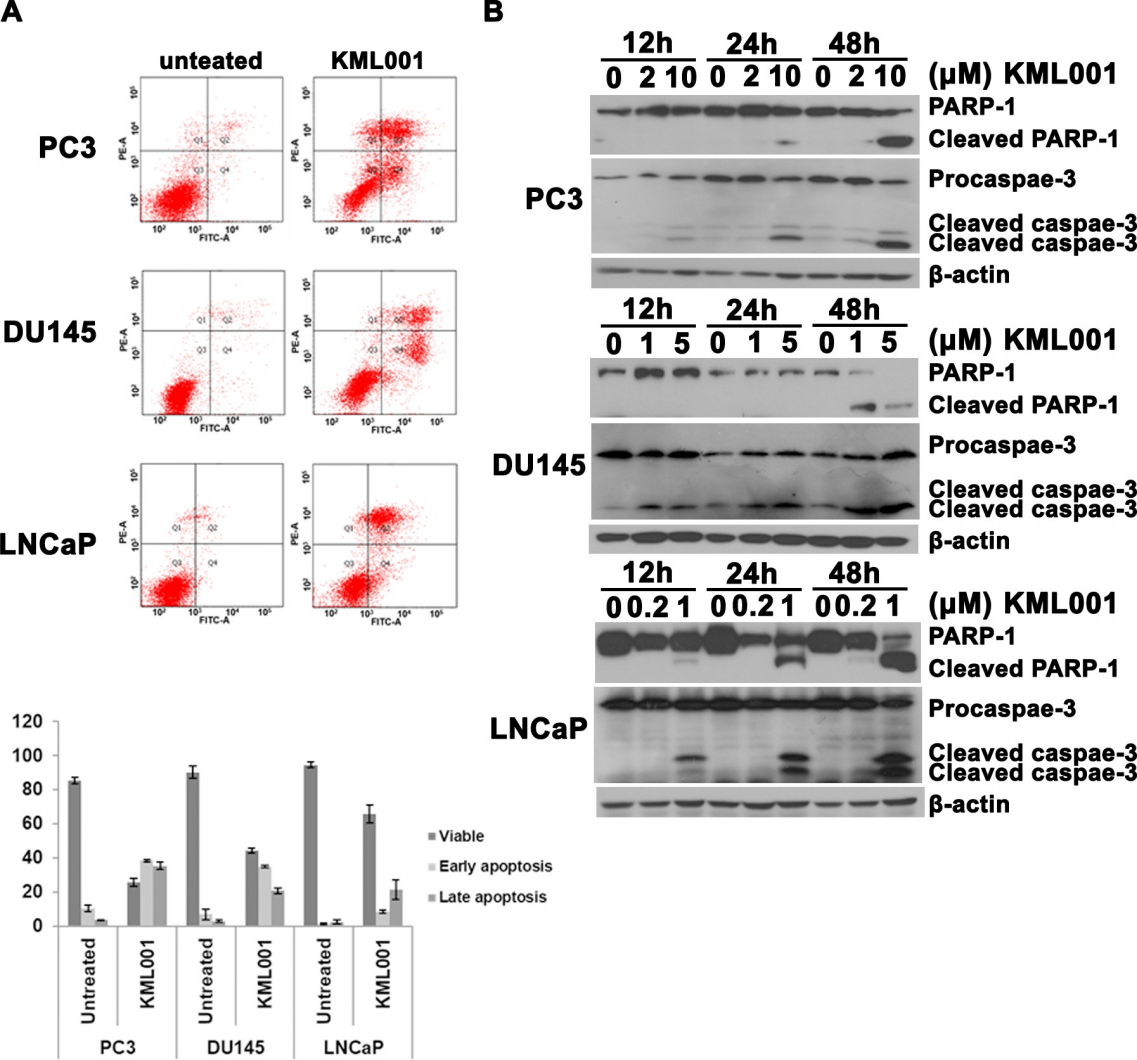
Induction of apoptosis by KML001 in prostate cancer cells. (A) FACS analysis of annexin V/PI staining. Results show early apoptosis, defined as annexin V-positive and PI-negative cells, and late apoptosis, defined as annexin V-positive and PI-positive cells. Results were expressed as means ± SD of three independent experiments. (B) Western blot analysis of the time- and dose-dependent cleavage of PARP and activation of procaspase-3.

There is an error in Fig 4, “Induction of autophagy by KML001 in prostate cancer cells.” Please see the correct Fig 4 here.

**Fig 4:**
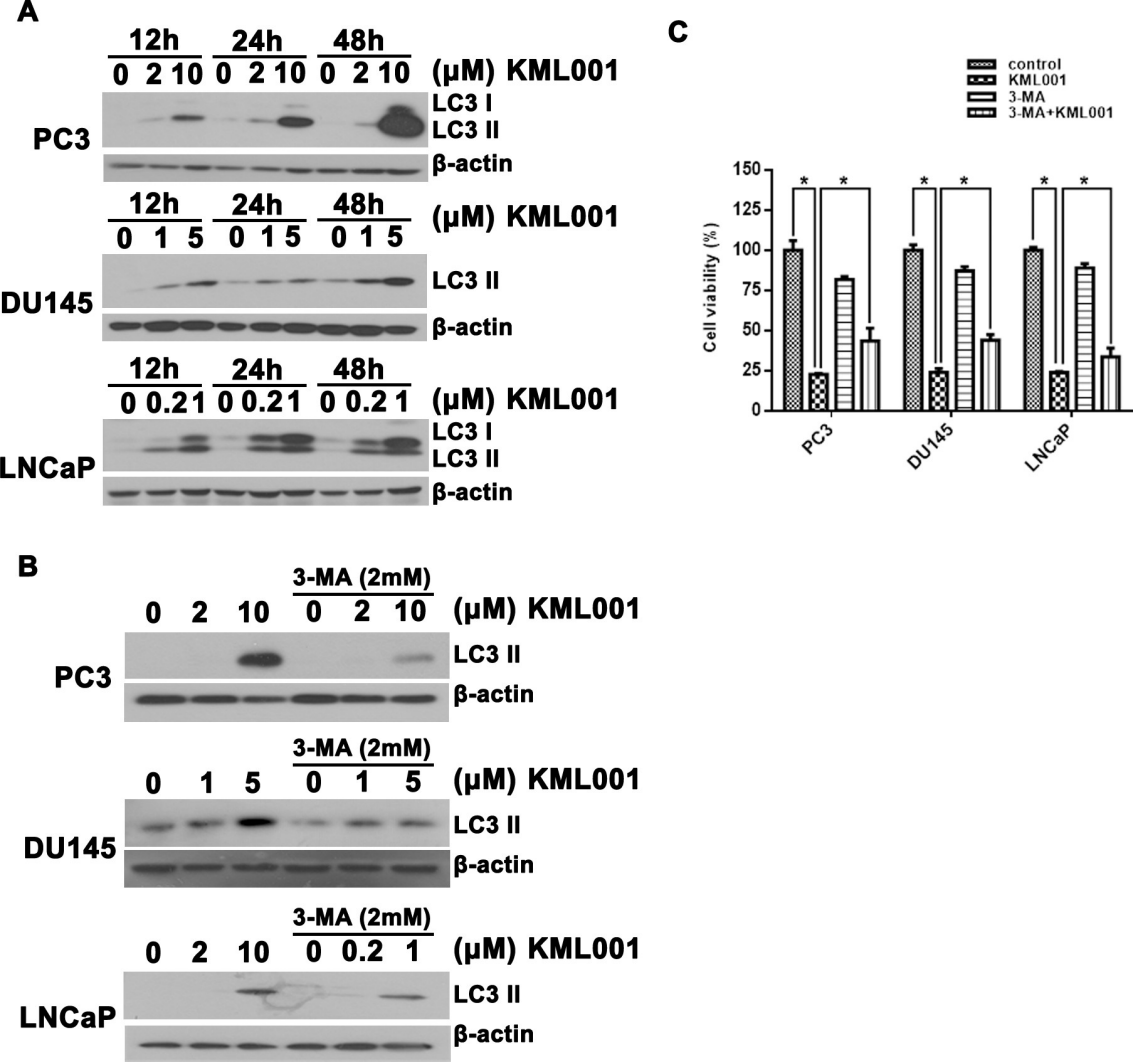
Induction of autophagy by KML001 in prostate cancer cells. (A) Western blot analysis of the time- and dose-dependent conversion of LC3-I to-II. (B) Inhibition by 3-MA of KML001-induced conversion of LC3 in prostate cancer cells. (C) Cells were exposed to 10 μM (PC3), 5 μM (DU145), or 2 μM (LNCaP) KML001 in the presence or absence of 1 mM 3-MA for 72 h. Results were expressed as means ± SD of three independent experiments. * p < 0.05 by one-way ANOVA.

There is an error in Fig 5, “Regulation of autophagy and apoptosis by ROS.” Please see the correct Fig 5 here.

**Fig 5:**
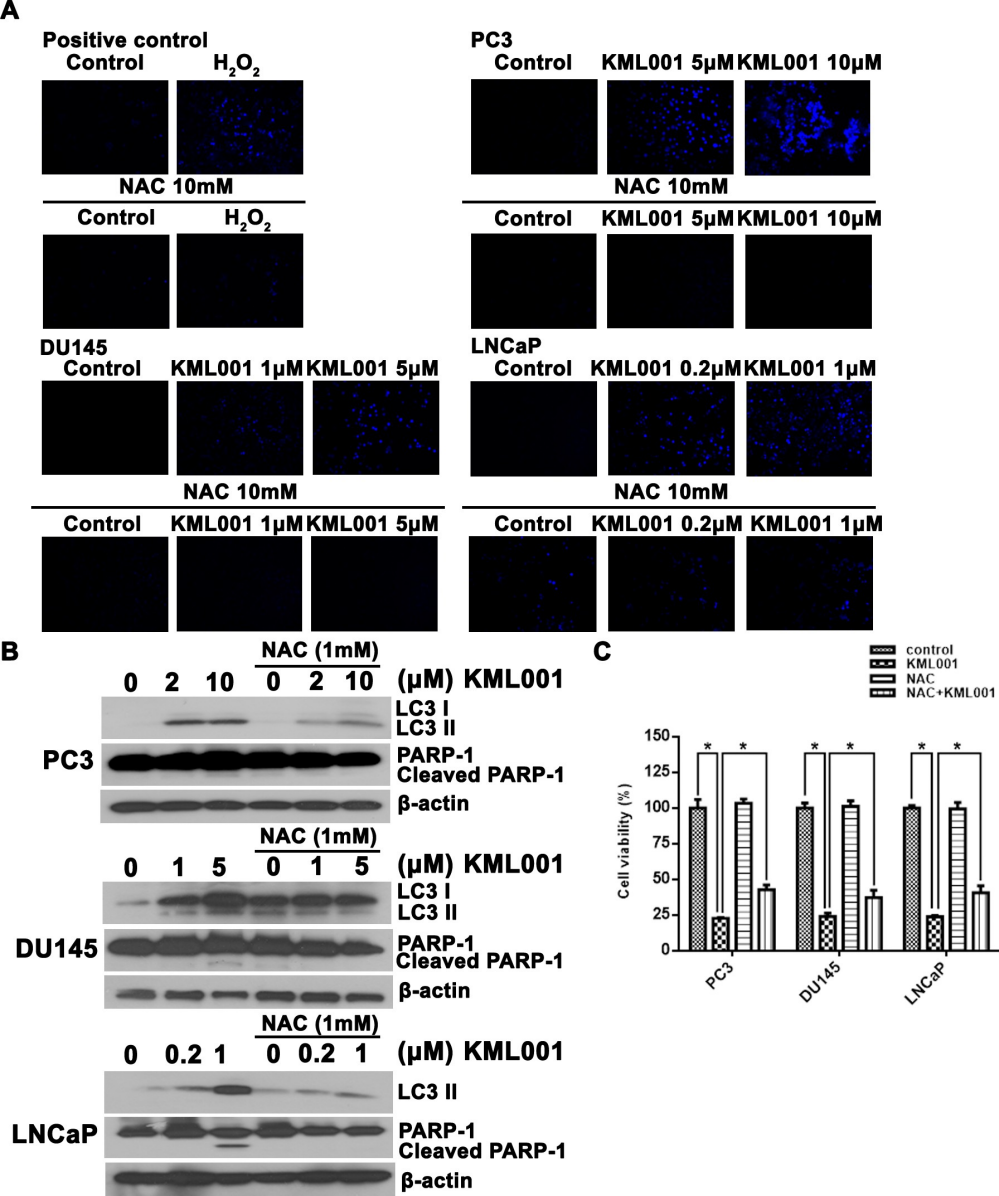
Regulation of autophagy and apoptosis by ROS. All 3 prostate cancer cells were treated with the indicated concentration of KML001 in the absence or presence of 5 mM NAC for 24 h. (A) KML001 induces dose-dependent ROS (blue) accumulation. Cells were stained with DCFH-DA and washed with PBS. More than three fields in each cell were observed by fluorescence microscope (200×), and representative images are shown. (B) NAC inhibition of KML001-induced conversion of LC and caspase activation in prostate cancer cells. (C) Cells were exposed to 10 μM (PC3), 5 μM (DU145), or 2 μM (LNCaP) KML001 in the presence or absence of 1 mM NAC for 72 h. Results were expressed as means ± SD of three independent experiments. * p < 0.05 by one-way ANOVA.
